# Machine Learning analysis of high-grade serous ovarian cancer proteomic dataset reveals novel candidate biomarkers

**DOI:** 10.1038/s41598-022-06788-2

**Published:** 2022-02-23

**Authors:** Federica Farinella, Mario Merone, Luca Bacco, Adriano Capirchio, Massimo Ciccozzi, Daniele Caligiore

**Affiliations:** 1 Division of Clinical Pathology, Laboratori Vita s.r.l., Via Sabaudia 19, 04100 Latina, Italy; 2 Unit of Computer Systems an Bioinformatics, Department of Engineering, Università Campus Bio-Medico di Roma, Via Alvaro del Portillo 21, 00128 Rome, Italy; 3 ItaliaNLP Lab, Istituto di Linguistica Computazionale “Antonio Zampolli”, National Research Council, Via Giuseppe Moruzzi, 1, 56124 Pisa, Italy; 4 Computational and Translational Neuroscience Laboratory, Institute of Cognitive Sciences and Technologies, National Research Council (CTN-ISTC-CNR), Via San Martino della Battaglia 44, 00185 Rome, Italy; 5 AI2Life s.r.l., Innovative Start-Up, ISTC-CNR Spin-Off, Via Sebino 32, 00199 Rome, Italy; 6 Unit of Medical Statistic and Epidemiology, Università Campus Bio-Medico di Roma, Via Alvaro del Portillo, 21, 00128 Rome, Italy; 7 Webmonks s.r.l., Via del Triopio, 5, 00178 Rome, Italy

**Keywords:** Diagnostic markers, Biomedical engineering, Tumour biomarkers

## Abstract

Ovarian cancer is one of the most common gynecological malignancies, ranking third after cervical and uterine cancer. High-grade serous ovarian cancer (HGSOC) is one of the most aggressive subtype, and the late onset of its symptoms leads in most cases to an unfavourable prognosis. Current predictive algorithms used to estimate the risk of having Ovarian Cancer fail to provide sufficient sensitivity and specificity to be used widely in clinical practice. The use of additional biomarkers or parameters such as age or menopausal status to overcome these issues showed only weak improvements. It is necessary to identify novel molecular signatures and the development of new predictive algorithms able to support the diagnosis of HGSOC, and at the same time, deepen the understanding of this elusive disease, with the final goal of improving patient survival. Here, we apply a Machine Learning-based pipeline to an open-source HGSOC Proteomic dataset to develop a decision support system (DSS) that displayed high discerning ability on a dataset of HGSOC biopsies. The proposed DSS consists of a double-step feature selection and a decision tree, with the resulting output consisting of a combination of three highly discriminating proteins: TOP1, PDIA4, and OGN, that could be of interest for further clinical and experimental validation. Furthermore, we took advantage of the ranked list of proteins generated during the feature selection steps to perform a pathway analysis to provide a snapshot of the main deregulated pathways of HGSOC. The datasets used for this study are available in the Clinical Proteomic Tumor Analysis Consortium (CPTAC) data portal (https://cptac-data-portal.georgetown.edu/).

## Introduction

Ovarian cancer is the seventh most common cancer in women and the eighth-most common cause of cancer death overall, with five-year survival rates below 45%. Along with the increasing life expectancy, the number of cases diagnosed each year is also growing, with only a minimal improvement in mortality^1,2^.

Although once considered a single entity, ovarian cancer can be subdivided into different histological subtypes that differ in molecular patterns, cells of origin, and clinical features. Among these types, high-grade serous ovarian carcinoma (HGSOC) is the most commonly diagnosed^3^ and is responsible for an elevated number of deaths. Its molecular features consist of a p53 mutation for 96% of the cases, while BRCA1/BRCA2 accounts for 22% of cases^4^. One of the principal factors influencing the elevated mortality of HGSOC patients is the inability to perform an early diagnosis, due to the symptoms being diverse and non-specific^5^. While the long-term survival of patients with stage I and II of ovarian cancer is respectively up to 90% and 70%, 4/5 of patients with HGSOC are diagnosed during stage III, and IV, resulting in a significantly lower survival rate of less than 20%^6,7^. Several studies have shown the importance of an accurate pre-operative evaluation and correct staging to enhance the prognosis of patients with a pelvic mass suspected of HGSOC. In fact, those treated by gynecologic oncologists had significantly lower morbidity and overall increased survival than those treated by general gynecologists and general surgeons^5, 8–10^.

Several biomarkers, such as CA125^11^, HE4^12^ and osteopontin^13^ have been used for the risk assessment of ovarian cancer in patients with a pelvic mass. Each of the biomarkers can be used alone or combined in multiple-biomarker algorithms (e.g. RMI^14^, ROMA^15^, OVA1^16^), having received both FDA and EU approval ^17^.

However, the screening methods based on these multiple-biomarker algorithms show different limits hampering their usage in clinical practice. All of them include CA125, a marker expressed in only 80% of Ovarian Cancer cases, and only in the 50% in the early stage of the disease^18^. The lack of expression in CA125 levels exhibited in some ovarian cancer cases and especially in the early stages of the disease is reflected by the sensitivity of the algorithms based on CA125. Furthermore, other studies show that different physiological and pathological conditions exhibit an increased expression of CA125 levels, thus limiting its specificity for the detection of this disease^19,20^. The use of additional biomarkers to overcome the limits of CA125 usually improves the sensitivity of the algorithm but always leads to a reduced specificity to detect ovarian cancer^21–23^. Hence, the necessity to find new molecular distinctive features that could both improve the disease understanding and be used as a starting point to develop new diagnostic tools, in order to establish one of the most appropriate treatment strategies, with the intention to improve ovarian cancer survival rates.

With this in mind, the purpose of this study was to dissect the pathways deregulated in HGSOC and find new possible biomarkers with high discriminating power, sensitivity and specificity that are localized in the serum, in order to be potentially assessed without invasive or expensive approaches. To reach this goal, we analyzed a publicly available ovarian cancer proteomic dataset using Machine Learning based algorithms, which can manage optimally such large scale omic datasets. The data used in this publication were generated by the Clinical Proteomic Tumor Analysis Consortium (NCI/NIH) ^24^.

Our computational approach allows us to overcome the decline in the specificity of existing tests, maintaining both sensitivity and specificity respectively at 98.2% and 97.2%.

## Materials and methods

### Database

For this study, we used the publicly available database generated by the Clinical Proteomic Tumor Analysis Consortium (CPTAC) ^24^. The Decision Support System (DSS) was trained, tested, and validated using the CPTAC Ovarian Cancer Confirmatory Study Proteomic Dataset, which includes the analysis form Ovarian tissue sample from a cohort of 100 individuals with HGSOC and 25 Non-Tumor ovarian samples, performed by the Johns Hopkins University (JHU) and Pacific Northwest National Laboratory (PNNL) using isobaric Tags for Relative and Absolute Quantification (iTRAQ) protein quantification method^25^. Clinical features were present only for Tumor patients. The Tumor cohort was composed of women ranging from 36 to 85 years, with an average age of 59. The 7% of the participants had an history of other malignancies. The anatomic site of origin of tumor specimens are: ovary 52%, omentum 41%, peritoneum 3%, pelvic mass 3% and unknown origin 1%. All samples are classified as “Serous Adenocarcinoma”. FIGO staging ranges from IIB to IV (not specified whether A or B), with the majority of the samples classified as stage IIIC (63.8%), followed by IV (15.2%), IIIB (7.6%), IIIA (2.9%), IC (1.9%), IIB (1%) and a remaining 7.6% of specimens having uncertain classification. The 80.8% of the samples are classified as Grade 3, 5.8% as Grade 2, 0.9% as Grade 1, while for 12.5% of the samples grading was not reported. The efficacy of the DSS was further tested on the dataset generated from the CPTAC and TCGA Cancer Proteome Study of Ovarian Tissue, including the analysis of samples from 174 Ovarian tumors, of which 169 from HGSOC, also performed by JHU and PNNL using iTRAQ^26^. Cohort is composed of women ranging from 35 years to 87, with an average age of 60.5. Tumor tissue site is Ovary for 98% of the samples, Omentum in 1% of the samples and Peritoneum ovary in 1%. All samples are classified as “Serous Cystadenocarcinoma”. FIGO staging of the samples goes from stage IC to IV (not specified whether A or B), where stage IIIC accounts for 69.9% of the samples, IV for 17%, IIIB and IIC accounting each one for 4.4%, IC for 1.5%, and IIA, IIB and IIA accounting each one for 1%. The 81.5% of the samples are Grade 3, 16.5% are Grade 2, 1% are Grade 1, while grading is unknown for 1% of the samples. Datasets were subsequently processed in Python (distribution 3.9.1) using NumPy and pandas libraries to merge JHU and PNNL datasets and remove protein columns containing more than 10% of missing values. After that, the data were processed and analyzed using a software tool coded in MATLAB2020b (Mathworks Inc., MA).

### Machine Learning pipeline

**Figure 1:**
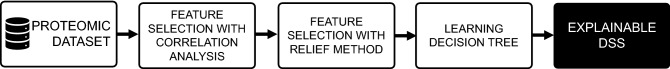
Machine Learning pipeline.

Here we describe the Machine Learning pipeline used to develop the Decision Support System. Each sample from the dataset is described by its features (i.e., the proteins). We report such pipeline in Fig. 1. It includes the following steps:

#### Feature selection based on correlation analysis

In this step, we computed for each feature the Pearson correlation coefficient with respect to the target variable (tumor/non tumor). The correlation coefficient between two random variables is a measure of their linear dependency. If each feature has N scalar observations, then the Pearson correlation coefficient of the *i*-th feature $f_{i}$ is defined as

1 $$\begin{aligned} \rho ({f_{i},t})= \sum _{j=1}^N\left( \frac{f_i(j)-\mu _{f_{i}}}{\sigma _{f_{i}}}\right) \left( \frac{t(j)-\mu _{t}}{\sigma _{t}}\right) \end{aligned}$$

where $\mu _{f_{i}}$, $\sigma _{f_{i}}$, $\mu _{t}$, $\sigma _{t}$ are the mean and standard deviation of the *i*-th feature and the target variable, respectively. The values of the coefficients can range from − 1 to 1, with − 1 representing a direct, negative correlation, 0 representing no correlation, and 1 representing a direct, positive correlation. All features with an absolute value of the correlation coefficient higher than 0.6 are then selected. In this way, we selected all the features with a high (positive or negative) correlation with the target variable.

#### Feature selection based on relief method

All the features selected from the Correlation Analysis are then examined with a second feature selection step based on the ReliefF algorithm^27^. Such an algorithm ranks the importance of the features with respect to the target value. The importance of a feature is represented by the weight of that feature. The values of those weights can range from $-1$ to 1, with the largest positive weights assigned to the most important features. The algorithm penalizes the features that provide different values to *k* neighbors of the same class while rewarding the ones that provide different values to *k* neighbors of different classes.

#### Decision tree

The features (i.e. the proteins) selected by the reliefF method are used to train the CART^28^ algorithm for the binary (Tumor/Non-Tumor) classification task. We chose to use a decision tree classifier for its high interpretability and explainability, unlike other methods of machine and deep learning. The CART tree is a binary decision tree that is constructed by splitting a node into two child nodes repeatedly, beginning from the root node that contains the whole learning sample. The basic idea of the tree growth is to choose a split among all the possible splits at each node so that the resulting child nodes are the “purest”. The purity metric defines a node as 100% impure when its samples evenly belong (50:50) to both the classes while defining a node as 100% pure when all of its data belongs to a single class. In this algorithm, only univariate splits are considered. That is, each split depends on the value of just one feature. At node *t*, the best split *s* is chosen to maximize a splitting criterion $\Delta i(s,t)$. When the impurity measure for a node can be defined, the splitting criterion corresponds to a decrease in impurity. In our case, we used a Gini criterion as the impurity measure. During the training, we chose not to impose a control on the tree’s depth, fixing the maximum number of splits as the size of the training set $-1$ and the minimum leaf size (the minimum number of samples in the leafs) as 1. Furthermore, we fixed the cost of classifying a sample into class *j* if its true class is *i* equal to:

- $$C_{i,j} = 1$$, if $$i \ne j$$
- $$C_{i,j} = 0$$, if $$i = j$$

We decided also not to implement a pruning strategy.

### Performance evaluation

To evaluate the performance of our system we computed the confusion matrix. A confusion matrix is an N $\times$ N matrix used for evaluating the performance of a classification model, where N is the number of target classes. In our case, the task performed by the model is a binary classification task, thus N is equal to 2. From the confusion matrix we calculated the classification accuracy $\left( Acc=\frac{TP+TN}{P+N}\right)$, the precision per class $(P_{Tumor}=\frac{TP}{TP+FP}$ and $P_{Non Tumor}=\frac{TN}{TN+FN })$, sensitivity and specificity $\left( Sensitivity=\frac{TP}{P},\, Specificity=\frac{TN}{N}\right)$. Furthermore for each class we compute the F1 score, a relevant metric in case of unbalanced dataset, $F1_{Tumor}=2*\left( \frac{P_{Tumor}*Sensitivity}{P_{Tumor}+Sensitivity}\right)$ and $F1_{NonTumor}=2*\left( \frac{P_{Non Tumor}*Specificity}{P_{Non Tumor}+Specificity}\right)$.

As usual, P and N denote the number of positive patients (with Tumor) and negative patients (Non-Tumor) records, whereas TP, TN, FP and FN stands respectively for true positive, true negative, false positive and false negative classifications. A true positive classification implies that the patients are correctly detected by the system as patients without tumor, whereas a true negative classification indicates that the system correctly recognizes the patients with HGSOC. We developed two main performance test:

- *Test 1* This test is developed to evaluate the performance of the system only on CPTAC dataset using a 5-fold cross-validation procedure as follows. First, we randomly shuffled the dataset and split it into 5 groups. For each group, a single group is taken as a hold out or test data set and the remaining groups as a training data set. After training and test, the evaluation score is retained and the model is discarded. This operation is then repeated for each group. Importantly, each sample in the data set is assigned to an individual group and stays in that group for the duration of the procedure. This means that each sample is given the opportunity to be used in the hold out set once and used to train the model 4 times. This procedure results in a less biased or less optimistic estimate of the system performance than other methods, such as a simple train/test split.
- *Test 2* This test is developed to evaluate the robustness of our system. We trained the system on CPTAC Dataset and tested it on a different dataset called Cancer Proteome Study of Ovarian Tissue (TCGA). This latter dataset is composed of 216 tumor patients.

### Pathway enrichment analysis

We used the ranked lists of proteins resulting from the correlation analysis, as input to perform a Pathway Enrichment Analysis using GSEA^29,30^ v.4.1.0 desktop software. The pathway gene set database was: Human_GO_AllPathways_with_GO_iea_January_13_2021_symbol.gmt release 13-01-2021, downloaded from http://baderlab.org/GeneSets. This file includes pathways from GO, Panther, NetPath, NCI, Reactome and MSigDB, both C2 and Hallmark collection. The number of permutations was set to 1000 and the maximum size of the sets was set to 200. Visualization of enrichment results was performed with Cytoscape^31^ v.3.8.2 using EnrichmentMap Pipeline Collection apps^32^, setting the FDR Q value cutoff to 0.01. In this work, we selected all the features with a coefficient higher than the average value taken by the positive coefficients.

## Results

As the first step of feature selection, the correlation was assessed between each feature and the tumor or non tumor variable, in order to possibly identify the most relevant molecular features of the tumor phenotype. The dataset after the pre-processing step consisted of 209 samples and 6223 proteins. In Table 1 we reported the results obtained setting the correlation coefficient cutoff to 0.6, thus reducing the significant features to 137 proteins. After the second step of feature selection, the list was further reduced to 46 proteins.

**Table 1:** Here are summarized the results of the correlation between proteomics data and tumor phenotype. It appears that a vast portion of the proteins displayed no evident correlation, and the majority of the proteins were negatively correlated.

	Tumor
Positive correlation	20
Negative correlation	117
Noncorrelation	6086

We then used the entire set of proteins and their respective correlation coefficient as a ranked list to perform a GSEA pathway enrichment analysis. The output was subsequently visualized and interpreted using the Cytoscape add-on EnrichmentMap. Resulting Normalized Enrichment Scores (NESs) ranged from -3.3251 to 3.4016. A subnetwork (Fig. 3) was generated from the main enrichment map selecting the most enriched pathways, setting the cutoff of NES to $+-$ 2.5, in order to drive the attention only on the most represented pathways. As in Fig. 3A, B the over-represented pathways are related to three main categories: RNA maturation and export, Translation and DNA Repair. By contrast, under-represented pathways (Fig. 3C) include: immune response, cell-matrix adhesion and extracellular matrix adhesion, protease activities, G-Protein coupled receptors signalling, myogenesis, muscular contraction, wound healing and blood coagulation.

### Explainable decision support system for tumor/non-tumor classification and biomarker discovery

**Figure 2:**
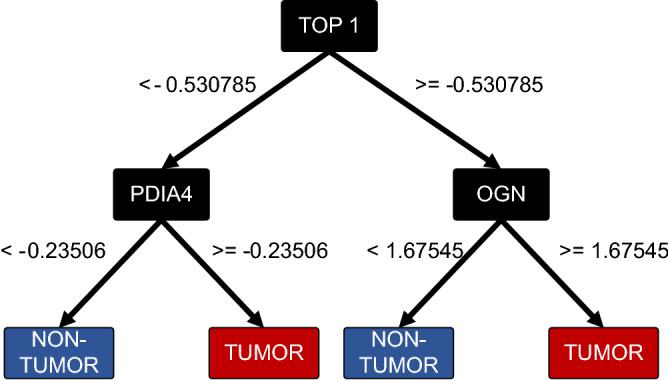
Final decision tree, with focus on the biomarkers.

With respect to test 1, we evaluated our method on the dataset presented in “Database” section. So, we started with a full dataset consisting of 209 samples and 6223 proteins. After the first step of Feature Selection based on Correlation Analysis, 137 features were left. Then, after the ReliefF-based Feature Selection step, we obtained 46 proteins. Finally, the dataset comprising 209 samples of 46 features was used to train the decision tree classifier. The model and the biomarkers achieved are shown in Fig. 2. The model is characterized by a graph with split conditions on three proteins: TOP1, PDIA4 and OGN. Furthermore, in Table 2 we report the classification confusion matrix that was computed collecting the prediction at the end of each iteration of the 5-fold cross-validation. All computed metrics from the confusion matrix are equal to 98.1% for accuracy, 98.2% for the sensitivity, 97.6% for specificity, 93% for precision of Non-Tumor class and 99.4% for precision of Tumor class, and 95.3% and 98.8% for F1-score of Non-Tumor and Tumor classes, respectively. With respect to test 2 we analyze the robustness of our system: for this reason we trained it on a dataset (CPTAC) and tested on a different one (TCGA). This latter dataset is composed of 216 tumor patients. In Table 3 we report the confusion matrix achieved. Furthermore, we calculate the accuracy of the system and the precision, sensitivity and F1-score per Tumor class that are equal to 98.2%, 100%, 97.2%, and 98.6% respectively. We did not computed metrics regarding the Non-Tumor class since the TCGA dataset does not present samples of this class.

**Table 2:** This Confusion Matrix is achieved in fivefold-cross-validation on CPTAC Ovarian Cancer Confirmatory Study Proteomic Dataset (209 samples). The matrix compares the actual target values (Truth) with those predicted (Pred.) by our model. On first diagonal are reported the samples correctly classified, whereas on second diagonal are reported the misclassified samples.

Pred.	Truth	
	Non-tumor	Tumor
Non-tumor	40	3
Tumor	1	165

**Table 3:** This Confusion Matrix reports the performance of our system trained on CPTAC Ovarian Cancer Confirmatory Study Proteomic Dataset and tested on TCGA Cancer Proteome Study of Ovarian Tissue (216 samples). The matrix compares the actual target values (Truth) with those predicted (Pred.) by our model. On first diagonal are reported the samples correctly classified, whereas on second diagonal are reported the misclassified samples. The TCGA dataset only presents samples from the Tumor class.

Pred.	Truth	
	Non-tumor	Tumor
Non-tumor	0	6
Tumor	0	210

## Discussion

Given the impact and the high mortality rate of HGSOC, numerous studies from the past few years took advantage of ’-omic’ scale expression data to characterize its underlying molecular features and to discover novel biomarkers. Nevertheless, the vast majority of existing studies makes use of RNA expression rather than protein expression. The main reason is the advantage of transcriptomics being a robust and cost-effective high-throughput technology. However, mRNA levels do not always correlate to protein abundance, given the number of regulatory processes occurring after mRNA transcription^33,34^. Hence, to find novel biomarkers suitable for cost-effective and non-invasive diagnostic methods such as blood or serum testing, we choose to base our analysis on Proteomics data.

### Correlation-based overview on the most deregulated pathways

**Figure 3:**
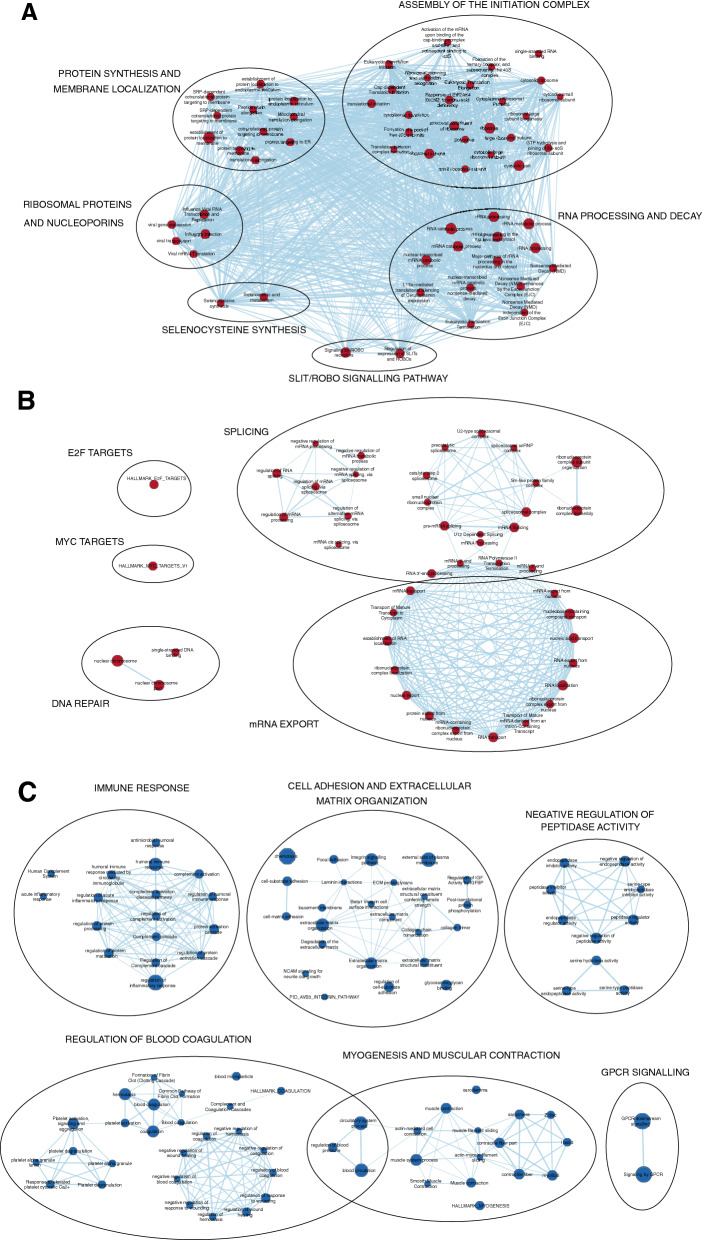
A Subnetwork was created from the main network to increase the interpretability. Red and blue nodes represent pathways that are upregulated (**A**, **B**) and downregulated (**C**). The diameter of each node is proportional to the number of proteins included. Pathways sharing proteins are connected with blue edges, with the thickness of the edges proportional to the number of protein shared. Clusters of nodes were manually annotated.

We first performed a correlation analysis. In this way, we reduced the number of features in the dataset, and at the same time, removed the “background noise” represented by the proteins that had a random correlation with the Tumor phenotype^35^. We then used the gene set enrichment analysis to extract biological insight from the ranked list of proteins that emerged from the correlation analysis. Among the over-represented pathways, displayed in Fig. 3 and summarized in Table 4, we found established and well-known cancer signatures, such as the increase of MYC and E2F downstream genes and DNA-Repair related genes such as MCMs and RAD21^36–39^. Interestingly, as shown in Fig. 3B, pathways related to mRNA splicing, export, metabolism, and translation were strikingly abundant and predominant among all the over-represented pathways. Given the crucial role of splicing as a source of biological complexity and plasticity, this same mechanism can be exploited by cancer cells to adapt and thrive in tumor-induced pathological conditions such as hypoxia^40^ and, favoring tumor progression, by contributing to the reprogramming of the cellular processes^41^. In accordance with this, a study shows that the spliceosome inhibitory drug Sudemycin is able to induce selective cytotoxicity in chronic lymphocytic leukemia (CLL) cells by targeting SF3B1, a component of U2 snRNP, which is also found in 13 nodes of our network. At the level of RNA export, there are several forms of cancer associated with dysregulation of some nucleoporins (Nup98, Nup214), components of the transcription-export complex TREX (THOC1), and exportines (XPO1, XPO5) that are also included in several nodes of our network and may be worth investigating further for their involvement in HGSOC^42–44^. As shown in Fig. 3A a large portion of pathways involved in the assembly of the initiation complex and ribosome biogenesis were significantly over-represented. Increasing evidence links deregulation of translational control to cancer insurgence and progression. Indeed, one of the most regulated steps during translation is its initiation, given its role in the decision of the rate of production of every protein, or if it is produced at all^45^. It is therefore not surprising that initiation factor encoding genes (eIFs) are overexpressed in a variety of cancers, such as breast, prostate and pancreatic cancer^46, 47^. Altered ribosome biogenesis also concurs to the altered translational activity of cancer cells; for example, it has been observed that in the aggressive breast cancer cell line MA-, 43S pre-rRNA was abnormal, resulting in an impaired ability to initiate p53 cap-independent translation via IRES^48^. Another cluster of pathways that stood out from our analysis involves nonsense-mediated decay (NMD) activity. NMD is a mechanism of post-transcriptional gene regulation, whose main purpose is exerting quality control on the mRNA through the recognition of premature termination codons (PTC), that may be introduced because of genetic mutations, or errors occurring during transcription or splicing. Beyond quality control, NMD emerged also as a mechanism for fine-tuning the amount of certain proteins^49^. An example is represented by the regulation of selenocysteine-containing proteins (SePs), such as glutathione peroxidase 1 (Se-GPx1) abundance in response to a decrease in selenium (Se) concentrations via NMD recognition of a Sec TGA codon^50^. Indeed, among the pathways present in this highly interconnected cluster, two groups of proteins are involved in selenocysteine synthesis^51^. SePs are known to be oxidoreductases, using selenocysteine in their active site. Their role in malignancy progression may vary according to the stage: on one hand they can inhibit tumor development by dampening oxidative insults that could induce mutagenesis and genomic instability while, on the other, they could offer tumor cells a competitive advantage to oxidative stress and chemotherapeutics, at an advanced stage^52^. This may indicate that in the context of HGSOC, they could favor tumor progression. The last members of this supercluster are proteins involved in the Slit/Robo pathway. Slits are a family of secreted proteins, as they bind to the transmembrane Robo receptors, they activate a signalling pathway that regulates various physiological processes, such as neural axon guidance, angiogenesis, cellular proliferation and motility, thus making it worthwhile to lead future research toward investigating their role as new druggable targets for HGSOC^53, 54^. Conversely, Fig. 3C shows the pathways that are significantly less represented in tumor cells than expected in physiological conditions. The first recognizable cluster involves the immune response. The avoidance of immune destruction is one of the hallmarks of cancer and has always represented a hot topic for research since the discovery of immunotherapy focused on targeting immune checkpoints^55^. In particular, the central nodes are involved in the regulation of complement activation, suggesting that HGSOC cells counteract the complement activation also by downregulating proteins involved in its activation such as CR2^56^. The second cluster of Fig. 3C involves cell-substrate adhesion and extracellular matrix (ECM) organization. Under-representation of pathways related to adhesion is a characteristic of cancer cells, in fact, adhesion molecules not only maintain contact with other cells or the substrate but also play a role as signalling molecules for a variety of cellular functions, such as growth regulation and gene expression, moreover, loss of adhesion is related to the Epithelial-Mesenchymal Transition (EMT), which leads to cell migration and invasiveness^57,58^. Here we found that proteases inhibitor-related pathways are significantly underrepresented. Proteases are enzymes that catalyze the hydrolysis of proteins, they take part in a plethora of physiological functions and their deregulation is associated with as many pathologies such as neurodegenerative disorders, inflammatory diseases, cardiovascular diseases and cancer^59^. Serpins, in particular, are serine protease inhibitors, regulating several biological activities, including coagulation, regulation of blood pressure, angiogenesis and hormone transport. Among the Serpins present in the nodes of our networks, Serpin B1, Serpin B5 and Serpin B9 have been found to be associated to tumor suppression and increased overall survival in Colorectal Cancer, suggesting that they could exert the same role also in HGSOC^60–62^. The next cluster examined in Fig. 3C belongs to the pathways involved in the negative regulation of coagulation. Activated Protein C (APC) is One of the most recurrent proteins among the nodes, along with its interactors Thrombodulin (TM) and Endothelial Cell Protein C Receptor (EPCR). APC is a serine protease that acts as an anticoagulant by inhibiting thrombin formation when the latter is bound to TM. This function is enhanced by EPCR, which binds APC and presents it to the TM-Thrombin complex^63^. The role of these three proteins in tumorigenesis is supported by the observation that the decrease or loss in their expression is related to tumor progression and poor prognosis^64^. It is accepted that enhanced coagulation represents a risk factor for the development of metastasis, possibly due to the fact that thrombin may favor the adherence of cancer cells either to platelets and to endothelial cells^65^. Interestingly, pathways related to myogenesis and muscular contraction were also found significantly under-represented. Among the nodes, Dystrophin (DMD) and other muscular distrophy-associated proteins: dysferlin and calpain-3 are found ubiquitously. These proteins are well-known for their role in the Duchenne muscular dystrophy, however, a role in cancer pathogenesis is slowly emerging. In this respect, it has been observed that Duchenne muscular dystrophy mdx mouse model was prone to develop skeletal muscle-associated tumors and that the dystrophic muscle presented genomic instability in a tumor-like fashion both in the mouse model and in humans^66^. Furthermore, DMD has been found to be downregulated in several tumors affecting the nervous system, hematological malignancies, melanoma and carcinomas, including lung adenocarcinoma, prostate, colon and breast cancer^67^. Our results show that DMD has a strong negative correlation to the tumor phenotype ($-0.75$), thus suggesting that an altered DMD expression may play a relevant role in the pathogenesis of HGSOC. The last underrepresented pathway is the G Protein-coupled receptor (GPCR) signalling pathway. GPCRs are the largest family of transmembrane signal transduction proteins, involved in a variety of biological processes, ranging from neurotransmission to hormone release, tissue development and homeostasis. It is not surprising that their dysfunction leads to numerous diseases^68^. Among the GCPRs present in the nodes of our network, the most relevant are GNA13, GNAS, SHH, FZD3 and SMO. These proteins exhibit loss of function mutations in cancers such as diffused B-cell lymphoma, Burkitt’s Lymphoma and basal cell carcinoma^69^, suggesting a possible role as oncosuppressors also in HGSOC. Overall, this analysis offers a plausible overview of the relevantly deregulated pathways in HGSOC, with most the pathways already known to be related to tumor progression, and some that could represent new paths to explore, in order to dissect the mechanisms underlying this gynecological malignancy. Given these premises, it may be worth lead future researches on the emerged proteins and their link to HGSOC.

**Table 4:** Summary of the 100 top-most deregulated pathways, ranked by their NES values, selected from the pathways composing the Subnetwork in Fig. 3. Pathways are named according to their Gene Ontology name or their standard name. In the left column are listed the 50 pathways that are found to be less represented in HGSOC tumor biopsies, a lower NES score corresponds to a lower representation. The right column displays the 50 pathways that appear to be the most over represented. A higher NES score correspond to a higher over representation.

Less represented pathways		Over-represented pathways	
Pathway description	NES	Pathway description	NES
Regulation of vascular smooth muscle cell proliferation	− 1.8195	Pre-mRNA splicing	3.4016
Positive regulation of phospholipid metabolic process	− 1.818	mRNA Splicing	3.3727
Neutrophil chemotaxis	− 1.8175	Regulation of mRNA processing	3.3537
Positive regulation of lipid transport	− 1.8168	Cap-dependent translation initiation	3.2584
Positive regulation of protein kinase B signaling	− 1.8157	rRNA processing	3.2518
IGF1R signaling cascade	− 1.8154	rRNA processing in the nucleus and cytosol	3.2488
Allograft rejection	− 1.8151	Influenza viral RNA transcription and replication	3.2475
Positive regulation of transporter activity	− 1.8148	Influenza infection	3.2379
PID_IFNG_PATHWAY	− 1.8141	Major pathway of rRNA processing in the nucleolus and cytosol	3.2266
BIOCARTA_BIOPEPTIDES_PATHWAY	− 1.8141	L13a-mediated translational silencing of ceruloplasmin expression	3.2208
Regulation of heart rate	− 1.8134	Spliceosomal complex	3.2119
Tertiary granule lumen	− 1.8111	Viral gene expression	3.2043
PID_CXCR4_PATHWAY	− 1.8088	Eukaryotic translation initiation	3.1978
Negative regulation of small molecule metabolic process	− 1.8082	GTP hydrolysis and joining of the 60S ribosomal subunit	3.1919
Negative regulation of cell-substrate adhesion	− 1.8075	Regulation of mRNA splicing, via spliceosome	3.1886
Regulation of glucose transmembrane transport	− 1.8065	Cytosolic ribosome	3.1753
Monocarboxylic acid transport	− 1.8039	Ribosome	3.1709
Positive regulation of cholesterol transport	− 1.8038	Formation of a pool of free 40S subunits	3.1677
Gastrin signaling pathway	− 1.8037	Viral transcription	3.1615
Activation of MAPKK activity	− 1.8037	Ribosomal subunit	3.1467
Cortical cytoskeleton	− 1.8036	Structural constituent of ribosome	3.1382
Amine metabolic process	− 1.8035	Eukaryotic translation elongation	3.137
Negative regulation of cell projection organization	− 1.8027	Translational initiation	3.1285
PID_ERBB1_DOWNSTREAM_PATHWAY	− 1.8018	Peptide chain elongation	3.1255
Negative regulation of neuron projection development	− 1.8012	Regulation of RNA splicing	3.122
IRS-related events triggered by IGF1R	− 1.8001	SRP-dependent cotranslational protein targeting to membrane	3.1111
Growth factor receptor binding	− 1.7996	Nonsense mediated decay (NMD) independent of the exon junction complex (EJC)	3.1079
Regulation of reactive oxygen species biosynthetic process	− 1.799	Viral mRNA translation	3.1065
Neuronal system	− 1.7989	Eukaryotic translation termination	3.0998
Negative regulation of axonogenesis	− 1.7965	HALLMARK_MYC_TARGETS_V1	3.0941
Opioid signalling	− 1.7963	Response of EIF2AK4 (GCN2) to amino acid deficiency	3.0812
Cell–cell adhesion via plasma-membrane adhesion molecules	− 1.7957	Protein targeting to ER	3.0792
BIOCARTA_HER2_PATHWAY	− 1.7956	Nonsense mediated decay (NMD) enhanced by the exon junction complex (EJC)	3.0764
PID_ERBB1_RECEPTOR_PROXIMAL_PATHWAY	− 1.795	Nonsense-mediated decay (NMD)	3.0737
Phosphatidylinositol binding	− 1.7946	Catalytic step 2 spliceosome	3.072
Phosphatidic acid biosynthetic process	− 1.7934	Selenocysteine synthesis	3.0554
Granulocyte chemotaxis	− 1.7913	SRP-dependent cotranslational protein targeting to membrane	3.0479
Regulation of blood vessel endothelial cell migration	− 1.791	Establishment of protein localization to endoplasmic reticulum	3.0463
B cell receptor signaling pathway	− 1.7905	Regulation of expression of SLITs and ROBOs	3.0373
Monocarboxylic acid binding	− 1.7896	Cotranslational protein targeting to membrane	3.0326
Toll-like receptor cascades	− 1.7875	Nuclear-transcribed mRNA catabolic process, nonsense-mediated decay	3.0094
Regulation of calcium-mediated signaling	− 1.7874	Regulation of alternative mRNA splicing, via spliceosome	3.0092
Triglyceride metabolism	− 1.7864	Selenoamino acid metabolism	2.972
Multicellular organismal movement	− 1.7857	Protein localization to endoplasmic reticulum	2.9604
Hydrogen peroxide catabolic process	− 1.7848	Ribonucleoprotein complex assembly	2.9379
Negative regulation of cellular response to growth factor stimulus	− 1.7846	Ribonucleoprotein complex subunit organization	2.9292
Gamma carboxylation, hypusine formation and arylsulfatase activation	− 1.7846	Activation of the mRNA upon binding of the cap-binding complex and eIFs, and subsequent binding to 43S	2.9227
Regulation of sodium ion transport	− 1.7843	rRNA processing	2.9199
Detection of external stimulus	− 1.7843	mRNA Processing	2.9151
Regulation of Rho protein signal transduction	− 1.7842	Translation initiation complex formation	2.8601

### Decision support system based on three discriminating biomarkers

As shown in Fig. 1, the step following Correlation Analysis consisted in a second feature selection method based on Relief algorithm. This allowed a further reduction and a list of the most important features ordered by importance score. The topmost 46 features were used as input to train and develop the highly discriminating Decision Support System, which is able to distinguish a tumor from a Non-Tumor patient based on the differential expression of three proteins: Topoisomerase 1 (TOP1), Protein Disulfide Isomerase Family A Member 4 (PDIA4) and Osteoglycin (OGN) ,as displayed in Fig. 2. Strikingly, as assessed in Test 1, the system showed 97.6% of specificity, 98.2% of sensitivity on the CPATC Ovarian Cancer Confirmatory Study Proteomic Dataset,with an F1 score of 98.8% for the tumor class and 93% for the fewer cases belonging to Non-Tumor class, while once tested on the second dataset (Test 2), it showed 97.2% sensitivity and 98.6% F1 score, thus eliminating the risk that the good performance was due to overfitting. Furthermore, these three proteins also appear to have a serum localization, thus making them ideal candidates, after clinical validation, for the development of non-invasive tests. The first biomarker is TOP1, one of the six human topoisomerases, whose function is to unwind negative DNA supercoilings occurring during the events of replication ^70^. TOP1 is also known to play a role in the maintenance of genomic integrity, in fact, a decrease in TOP1 activity, due to low expression or lack of recruitment to chromatin by SMARCA4, may result in DNA damage and genomic breaks^71,72^. This is reflected by the upregulation of TOP1 in cancer cells, which undergo through replicative and transcriptional stress^73^. Given this crucial role, there are several FDA-approved drugs targeting TOP1. The most famous are the camptothecin alkaloid derivatives, which act by binding at the interface between the DNA and the topoisomerase^74^. The second biomarker, PDIA4, is one of the largest member of the Protein Disulfide Isomerases family (PDIs), which are known to mediate protein folding via either the formation or the breakage of disulfide bonds^75^. Other than its protein folding function, exerted when located in the endoplasmic reticulum, PDIA4 can also be present on the surface of the platelet, where it participates in thrombus formation^76^. It has been observed to be over-expressed in a cohort of Epithelial Ovarian Cancer (EOC) patients, where it was associated with disease progression and poor prognosis^77^, potential mechanisms involve the inhibition of apoptosis emerged in another study, where the over-expression of PDIA4 in tumor cells reduced caspase 3 and 7 activity favoring cell growth^78^, thus potentially enabling tumor resistance to therapy^79^. Lastly, OGN, a small leucine-rich proteoglycan (SLRP) protein. Its function is different in different cell types: in the extracellular compartment it is involved in collagen cross-linking, while in vascular smooth cells (VSMCs) and fibroblasts, a reduced expression leads to cellular proliferation. Its implications in tumor progression are quite recent but evident. For instance, OGN appears to be under the control of p53, and several studies show a reduction or lack of OGN expression in a variety of cancers, among which breast, colon, lung, ovarian and pancreatic cancer^80^. It has been observed in bladder cancer that ECRG4 promotes OGN expression by upregulating NFIC, preventing the activation of NF-KB downstream pathways, thus inhibiting cell proliferation and migration^81^.

Furthermore, in breast cancer, OGN seems to reverse epithelial to mesenchymal transition by repressing the PI3K/Akt/mTOR axis^82^. Overall, the DSS managed to identify, among the HGSOC proteome, three proteins that are known to be linked to tumorigenesis. In addition, the high sensitivity and specificity of these biomarkers for the distinction between tumor and Non-Tumor patients, coupled with the fact that they also appear to be localized in the serum, is promising for their possible clinical use for the diagnosis of HGSOC. It’s worth noting that in our analysis seral biomarkers CA125 and HE4 were found to not correlate with Tumor phenotype, and were consequently dropped at the fist step of the pipeline. This prevented us from performing a proper comparison, since the lack of correlation implies that if we build a classifier using only these two proteins, this will be with any probability unable to distinguish Tumor from Non Tumor samples if applied to our datasets.

## Conclusions

To summarize, we provided a reliable overview of the most relevant deregulated pathways in HGSOC, focusing mainly on those genes that were not related directly to HGSOC before, thus providing novel associations and new starting points for future researches. Furthermore, we developed a Decision Support System able to find three possible Biomarkers for the diagnosis of HGSOC. These three proteins are ubiquitous and exert their primary function in physiological conditions. However, a role for TOP1 as an oncogene has been already strongly suggested, being found upregulated in different types of tumors, including breast, liver and colorectal cancers ^83–86^. Indeed, several TOP1-targeting drugs have received FDA approval ^74,87,88^. The connection of PDIA4 and OGN with tumor progression is relatively recent, PDIA4 has been found overexpressed in a cohort of EOC patients, and associated with poor prognosis, cell gowth and resistance. On the other hand, a decrease in OGN expression was found in different types of cancers. This is coherent with the results of our dataset analysis, in which we found they showed a strong correlation with the tumor phenotype, with TOP1 and PDIA4 positively correlating and OGN being negatively correlated. Furthermore, the predictive efficiency of this system in considerably high in both of the tested datasets. Notwithstanding, further validation is crucial to support this in silico results, and, for a possible clinical use, further studies are needed to assess if the proportions of these biomarkers are maintained in the serum as they are in HGSOC biopsies. Finally, once clinically and experimentally validated, this pipeline could be easily applied to other tumor datasets for the purpose of discovering novel biomarkers and clinical predictors.

## Data Availability

The datasets analysed during the current study are available in the Clinical Proteomic Tumor Analysis Consortium (CPTAC) data portal repository (https://cptac-data-portal.georgetown.edu/).
